# Awake craniotomy for the resection of a Broca-sited cerebral cavernous malformation with a developmental venous anomaly using near-infrared indocyanine green video angiography

**DOI:** 10.3171/2019.7.FocusVid.18626

**Published:** 2019-07-01

**Authors:** Issael Ramirez, Paldor Iddo, Sergey Spektor

**Affiliations:** Department of Neurosurgery, Hadassah University Hospital, Jerusalem, Israel

**Keywords:** cavernoma, awake, DVA, motor speech cortex, Ojemann, video

## Abstract

Cerebral cavernous malformations (CCMs) are known to be angiographically occult malformations with low perfusion of blood flow.^5^ Near-infrared indocyanine green (ICG) video angiography allows for intraoperative observation and documentation of blood flow in large and small vessels.^2,4^

Developmental venous anomalies (DVAs) are thought to be the most common cerebral vascular abnormality.^2,3^ The opportunity to differentiate intraoperatively between normal veins and DVA draining veins might be useful in the event of a possible venous sacrifice. Coagulation of the DVA can lead to devastating consequences. ICG reliably demonstrates margins between CCM and the venous structures.^1,2^ For these reasons, we decided to use ICG video angiography in this patient.

The video can be found here: https://youtu.be/9MONn0GkO4U.

## Transcript

We present this video of an awake craniotomy for the resection of a Broca-sited cavernoma with a developmental venous anomaly using ICG green video angiography.

Patient with a history of seizures since more than 5 years that showed worsening on the episodes and enlargement of the cavernoma in follow-up.

In the MRI protocol T1+gadolinium we can see the berry appearance on the left insula, with a rim of signal loss due to hemosiderin. We can also appreciate the large DVA on the medial aspect of the cavernoma.

FLAIR protocol shows the hyperintensity, classic cavernoma appearance.

In the coronal T1+gadolinium protocol we can see again the lesion and the relationship with the big DVA.

Functional MRI shows us the areas activated during speech planning in orange. Notice how most of those areas are around the cavernoma.

1:25 As stated in the title, we chose to do an awake craniotomy, with laryngeal mask, phenytoin the day prior to the surgery to prevent per operatory seizures, local anesthetic on the surgical wound, and block on the Mayfield points.

On the right side you can see a 3D reconstruction of the craniotomy and patient positioning. This was made on supine position, head fixed on a Mayfield frame and tilted to the right side.

The position of the head was done in a way that allows us and the patient a clear view for the moment that anesthesia is suspended.

We did a small navigation-guided craniotomy and irrigated the dura with local anesthetic. At this point, anesthesia is suspended, and patient is awake, obeying commands, and completely oriented.

We opened the dura in a cruciform fashion; brain was relaxed. Using silk 1-0, we elevated the four points of the dura opening.

As the dura is elevated, brain is exposed and arachnoid dissection is started. We decided to perform a transsulci approach. The arachnoid is first dissected anteriorly. Navigation is again verified, and we open the arachnoid more posteriorly.

2:50 At this point, we proceed to insert the Ojemann to the operating field.

We started to stimulate the cortex. At this point, no positive response is evident. We continue to stimulate around the surgical field.

As we go to the posterior aspect, patient suffers from speech arrest. The patient becomes completely aphasic. We verified again speech arrest occurs. Motor verbal cortex is then identified. We placed a cottonoid over, and with what we were sure was the DVA at the left of the video, we proceed with the injection of the ICG green. You can follow the DVA going deep into the brain, and in front the cavernoma, as expected, is nonfluorescence to ICG green.

3:44 We can appreciate the flow of the vessels around the cavernoma and the DVA itself represented by the color green.

With the cavernoma identified, we start the dissection. We can see chronic blood coming out.

Placement of retractor allows us for a more comfortable working space.

With the dissection, our goal was to detach the cavernoma first of all away from the DVA.

A good plane is found following the periphery of the lesion. On the floor you can see normal brain; a very good detachment is obtained, using bipolar and microsuction.

As stated before, normal brain seen on the deep aspect of the dissection.

We then proceed to dissect the cavernoma from the DVA. This is done very carefully to avoid any unwanted complication. We had a good plane; continuous interaction is being carried out with the patient at all times.

Complete dissection of all the margins of the cavernoma is achieved. We now detach it from the last remaining part; this is the last stage.

5:55 Complete one-piece resection is completed.

ICG green is again injected and you can observe the patency of the DVA preserved, with a good flow.

We wash with isotonic warm saline solution; hemostasis is achieved. We approximated the borders and placed synthetic dura.

Bone flap is fixed with two craniofixes, Vycril 3-0 and Monocryl for the skin. We found no need to leave a drainage.

6:47 Twenty-four hours postsurgery CAT scan shows no signs of bleeding on the surgical bed.

Follow-up 3 months postoperative MRI protocol T1+gadolinium shows complete resection of the cavernoma. DVA is evidently preserved and patent.

Postop FLAIR protocol shows no remaining signs of the cavernoma.
